# Unspecific clinical manifestations of insulinoma found in preoperative testing: a case report and narrative review

**DOI:** 10.3389/fendo.2026.1802157

**Published:** 2026-09-21

**Authors:** Manqing Yu, Xinlu Ji, Pingping Zheng

**Affiliations:** 1 Department of Endocrinology, The Affiliated Hangzhou First People’s Hospital, The College of Medicine, Westlake University, Hangzhou, China; 2 The Affiliated Hangzhou First People's Hospital, The College of Medicine, Westlake University, Hangzhou, China

**Keywords:** hyperinsulinemia, hypoglycemia, imaging sensitivity, insulinoma, selective arterial calcium stimulation test

## Abstract

**Background:**

Insulinoma is a rare endocrine tumor that is clinically characterized by the Whipple’s triad and can be part of multiple endocrine neoplasia syndrome type 1 (MEN1). Most insulinomas are less than 2 cm, but the smaller the tumor diameter, the lower the imaging sensitivity. Here we present a case of recurrent asymptomatic hypoglycemia with unremarkable contrast-enhanced CT and MRI imaging that led to the delayed identification of an insulinoma. The tumor was localized with the invasive selective arterial calcium stimulation test (SACST).

**Case presentation:**

A 59-year-old female presented with asymptomatic hypoglycemia detected in preoperative screening on two occasions over the past year. She had no classic symptoms of hypoglycemia such as dizziness, weakness, blackouts, anxiety, palpitations, tremors, or profuse sweating. Detailed history taking revealed that she has had two episodes of sudden irritability, personality changes, and abnormal violent behavior that were relieved with food. After extensive endocrine hormone testing and imaging to exclude the possibility of MEN1, she was diagnosed with a 1.5-cm insulinoma in the pancreatic tail. Her blood glucose, insulin and C-peptide levels returned to normal after tumor excision.

**Conclusion:**

Hypoglycemia unawareness develops after recurring episodes of hypoglycemia and increases the risk of life-threatening hypoglycemia. This case highlights the importance of identifying the underlying cause of hypoglycemia and implementing timely intervention.

## Introduction

Non-diabetic hypoglycemia can be difficult to detect in patients who are asymptomatic or unaware of the myriad of nonspecific symptoms. Long-term, untreated hypoglycemia can result in cognitive deficits and can be life-threatening. Hypoglycemia can be due to a wide range of underlying conditions, including malnutrition, medication, concomitance with other diseases, and tumors. Clinically significant hypoglycemia can be identified by Whipple’s triad (1): signs or symptoms of hypoglycemia, such as nausea, confusion, tremor, sweating, palpitations, or hunger (2); low blood glucose levels measured in the laboratory at the time of symptoms (3); symptoms alleviated by increasing blood glucose levels. The most common cause of hypoglycemia in non-diabetic patients is endogenous hyperinsulinemia, whereby insulin levels remain elevated despite very low blood glucose levels (1, 2). Hence, early diagnosis of the source of endogenous hyperinsulinemia is critical to the management of hypoglycemia in non-diabetic patients to avoid long-term adverse outcomes (3).

Insulinomas are rare, insulin-secreting pancreatic neuroendocrine tumors (NET) with an incidence of 0.7 to four cases per million per year (4). There is ongoing debate on the optimal cutoff for blood glucose levels for the diagnosis of hypoglycemia, which ranges from <2.5 mmol/L (5) to <3.0 mmol/L (1) to <3.9 mmol/L (6). The Chinese medical guidelines define hypoglycemia as blood glucose <2.8 mmol/L in non-diabetic patients (7). Hypoglycemia due to endogenous hyperinsulinemia is diagnosed via plasma insulin ≥3 mIU/L (18 pmol/L), plasma C-peptide ≥0.2 nmol/L (0.6 ng/L), and/or plasma proinsulin >5.0 pmol/L (1, 4, 5, 8, 9). Most insulinomas are small (<2 cm), intrapancreatic, solitary, and indolent (4, 10, 11). Although widely used, both CT and MRI perform poorly, with sensitivities ranging from 35% to 82% and from 35% to 63%, respectively (12).

Approximately 5% to 10% of insulinomas occur as a component of multiple endocrine neoplasia type 1 (MEN1) (11, 13). MEN1 is a rare, autosomal dominant disorder characterized by a mutation in the *MEN1* gene and the co-occurrence of two or more tumors originating from the parathyroid gland, pancreatic islet cells, and/or anterior pituitary. Routine genetic testing for MEN1 is usually not recommended in patients with newly diagnosed insulinoma (4). Hence, functional and imaging tests of the parathyroid and pituitary are performed to exclude other concomitant tumors associated with MEN1. For example, primary hyperparathyroidism is found in approximately 90% of patients with MEN1.

Here we present a case of unspecific clinical manifestations of recurrent hypoglycemia documented during preoperative testing, with no definitive localization of the insulinoma on CT or MRI. The less commonly used selective arterial calcium stimulation test (SACST) confirmed the diagnosis.

## Case presentation

A 59-year-old female was referred to the endocrinology department because two sets of blood tests documented asymptomatic hypoglycemia. Both sets of blood tests were performed as part of preoperative testing prior to ophthalmologic surgery. Her fasting blood glucose levels were 2.83 mmol/L before surgery one year ago and 2.18 mmol/L before surgery on the other eye two days ago. She had no dizziness, weakness, blackouts, anxiety, palpitations, tremors, or profuse sweating. Her medical history was unremarkable aside from two episodes of sudden irritability without obvious triggers, characterized by a significant personality change and abnormal violent behavior that ceased after eating. These two episodes occurred outside of the hospital and were not accompanied by blood draws to verify hypoglycemia. During this period, her weight increased by up to 10 kg but had since returned to her previous weight. She had a history of hypertension with the highest blood pressure of 150/95 mmHg, and was managed with irbesartan 150 mg once a day. Her mother had a history of hypertension and type 2 diabetes, and there was no other known family medical history. Physical examination was unremarkable.

The patient first underwent an endocrinology workup to determine whether hypoglycemia is caused by abnormal insulin production and secretion. The oral glucose tolerance test (OGTT) showed a normal fasting blood glucose level of 3.74 mmol/L and high maximum insulin and C-peptide levels of 57.97 mIU/L and 2.35 nmol/L, respectively (**Table 1**). The patient had a habit of frequent late-night food intake, which might explain the euglycemia during the fasting state. The OGTT results allowed the differential diagnosis of diabetes mellitus and reactive postprandial hypoglycemia.

**Table 1:** Oral glucose tolerance test results.

Timepoint	0 h	0.5 h	1 h	2 h	3 h
Blood glucose (mmol/l)	3.74	5.49	7.15	6.42	4.14
Blood insulin (mIU/L)	8.93	42.44	57.97	46.24	20.90
C-peptide (nmol/L)	0.57	1.46	2.14	2.35	1.75

Then, a 72-hour fasting test was initiated at 10 pm, but the patient was found to have hypoglycemia during baseline blood sampling at 8 am the next morning and at 15 min later during repeat sampling. At baseline, fingertip glucose was 3.1 mmol/L, blood glucose was 2.8 mmol/L, insulin was 15.26 mIU/L, and C-peptide was 0.86 nmol/L. Fifteen minutes later, fingertip glucose was 2.7 mmol/L, blood glucose was 2.52 mmol/L, insulin was 13.67 mIU/L, and C-peptide was 0.82 nmol/L. The 72-hour fasting test was terminated. Based on the current Chinese guidelines, there is no specified criterion for terminating the 72-hour fasting test (14, 15). Therefore, the initial diagnosis was hypoglycemia due to endogenous hyperinsulinemia, based on the diagnosis criteria in the medical guidelines: plasma glucose ≤2.8 mmol/L (50 mg/dl) (7), insulin level ≥3.0 mU/L (18 pmol/L), and C-peptide concentration ≥0.2 nmol/L (0.6 μg/mL) (1, 5).

The next step was to identify the cause of endogenous hyperinsulinemia. Contrast-enhanced CT and MRI of the upper abdomen both showed no obvious space-occupying lesion in the pancreas (**Figure 1**). Upon clinical correlation with the suspected insulinoma, the contrast-enhanced CT was reviewed again by radiology and was reported as showing a possible slightly enhanced nodule (4.93 mm × 9.50 mm) in the body of the pancreas during the arterial phase; an insulinoma could not be excluded.

**Figure 1:**
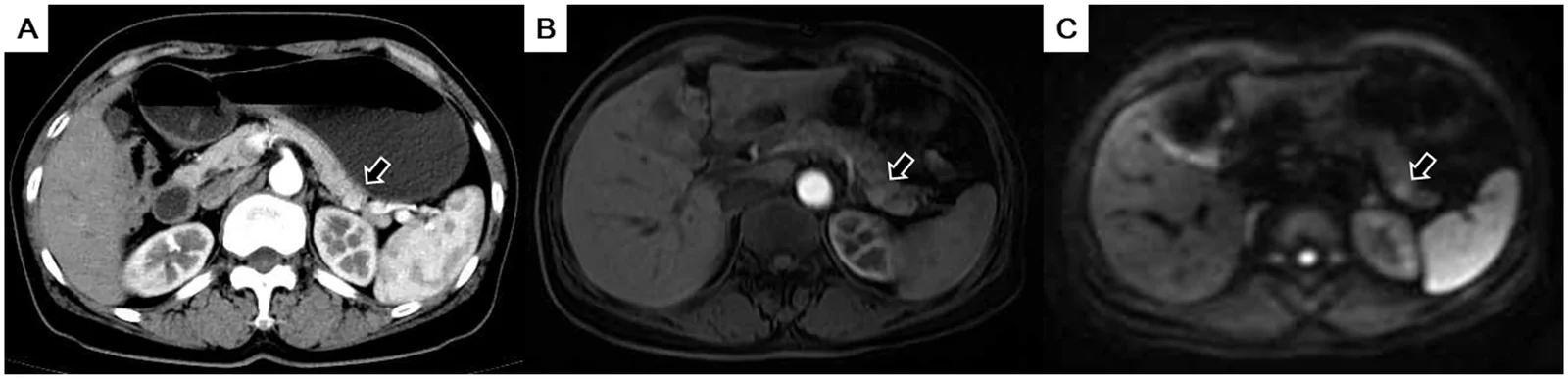
Imaging of the pancreas. **(A)** The contrast-enhanced CT was initially reported as showing no findings but was reviewed again and a possible slightly enhanced subcentimeter nodule was identified in the arterial phase. **(B, C)** The contrast-enhanced MRI was also initially reported as showing no findings, but upon retrospective review, a slightly enhanced nodule could be seen in the arterial phase **(B)** and with diffusion-weighted imaging **(C)**.

To identify the location of the insulinoma, a selective arterial calcium stimulation test (SACST) was performed (**Table 2**). A positive SACST is defined as a maximum insulin fold increase greater than 2. The sampled insulin secretion in the proximal segment of the splenic artery (SAP) was clearly abnormally elevated at >29-fold the baseline level, which indicated the location of the insulinoma (**Figure 2**).

**Table 2:** Pituitary axis and parathyroid functional test results.

Endocrine function	Test	Result	Normal range
Pituitary–adrenal	Blood ACTH (ng/L)	8AM: 18.20 4PM: 17.00 12AM: –	8AM: 10.0–90.0 4PM: 7.6–76.0 12AM: –
	Blood cortisol (ug/L)	8AM: 98.20 4PM: 90.60 12AM: 27.94	8AM: 43.0–224.0 4PM: 30.9–166.0 12AM: 43–224
Pituitary–thyroid	TSH (mIU/L)	3.822	0.6–4.8
	FT4 (pmol/L)	11.42	12.0–20.2
	FT3 (pmol/L)	4.65	4.0–6.2
	TT4 (ug/L)	62.4	54.39–115.0
	TT3 (ug/L)	0.9	0.71–1.43
Pituitary–gonadal	FSH (IU/L)	101.74	23–116
	LH (IU/L	35.41	5–52
	Prolactin (ug/L)	11.62	1.7–20
	Estradiol (pmol/L)	<43.31	0–113.8
	Progesterone (nmol/L)	0.74	0–2.32
	Testosterone (nmol/L)	0.64	0.24–1.25
Pituitary–growth hormone	Growth hormone (ug/L)	4.37	0.06–5
	IGF-1 (ug/L)	177	45–210
	IGF-1BP3 (mg/L)	5.16	3.4–9.5
Parathyroid	PTH pg/ml	27.43	15–65
	Calcium mmol/L	2.16	2.0–2.6
	Phosphate mmol/L	1.09	0.8–1.5

ACTH, adrenocorticotropic hormone; TSH, thyroid stimulating hormone; FT4: free thyroxine; FT3: free triiodothyronine; TT4: total thyroxine; TT3: total triiodothyronine; FSH: follicle-stimulating hormone; LH: luteinizing hormone; IGF-1: insulin-like growth factor 1; IGF-1BP3: insulin-like growth factor binding protein 3; PTH: parathyroid hormone.

**Figure 2:**
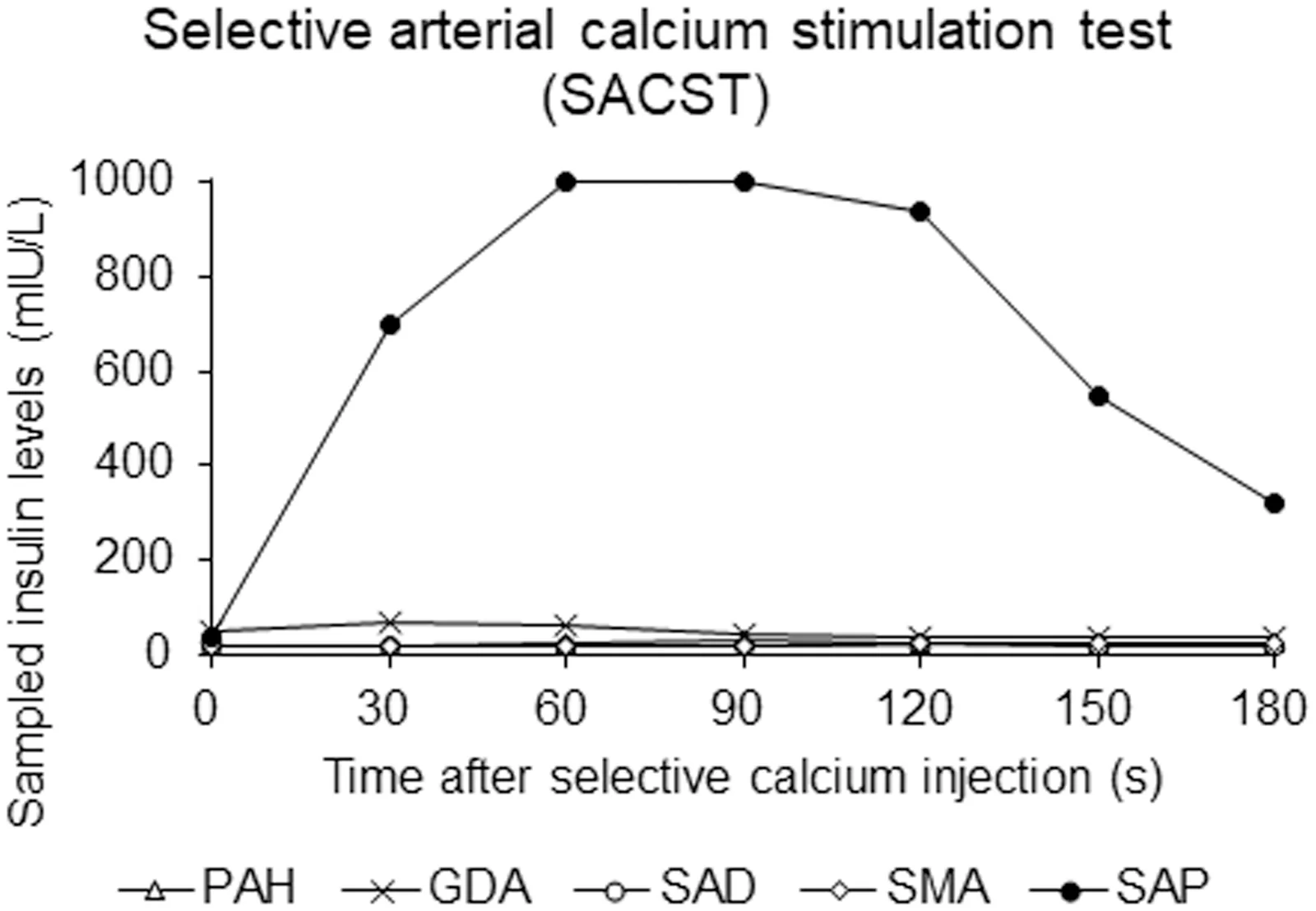
Selective arterial calcium stimulation test (SACST) results. The maximum fold increase in insulin levels compared with baseline insulin levels in the sampled hepatic vein after injection of calcium gluconate in the proper hepatic artery (PHA) was 1.68; in the gastroduodenal artery (GDA), it was 1.92; in the distal splenic artery (SAD), it was 1.54; in the superior mesenteric artery (SMA), it was 1.64; and in the proximal splenic artery (SAP), it was >29. The asterisk (*)denotes sampled insulin levels >1,000 mIU/L, which is above the maximum level of detection.

## Differential diagnosis

Insulin autoimmune syndrome (IAS) or Hirata’s disease was excluded by the absence of elevated anti-insulin autoantibodies (IAA), as measured by ELISA, at 5.12 IU/mL (normal range, 0 IU/mL–20 IU/mL).

Hypoglycemia due to diabetes mellitus was excluded by a normal OGTT and an HbA1c level of 5.1%.

The vast majority of insulinomas are sporadic, but they also can be associated with multiple endocrine neoplasia type 1 (MEN1 or Wermer syndrome), which manifests as a syndrome consisting of pituitary, parathyroid, and pancreatic tumors. Pituitary and parathyroid tumors were excluded based on imaging and functional tests according to current MEN1 guidelines (16, 17).

Pituitary tumors were excluded based on normal prolactin and IGF-1 levels, and contrast-enhanced pituitary MRI showed no obvious abnormality of the pituitary (16, 17). The functional tests of the pituitary axes showed no abnormality in cortisol, thyroid, reproductive, growth hormone, or parathyroid hormone levels (**Table 2**). Parathyroid tumors were excluded based on normal serum calcium, phosphorus, and parathyroid hormone levels (**Table 2**) (16, 17). Parathyroid ultrasound was performed to further exclude any parathyroid lesions and revealed cystic nodules around the lower poles of both thyroid glands, possibly representing parathyroid cysts.

To further exclude any other possible causes of hypoglycemia, such as immune-mediated hypoglycemia (18), autoimmune thyroiditis, and pancreatitis, the following tests were performed, and the results were negative: autoantibodies TgAb 96.4 IU/ml (0 IU/ml–4.5 IU/ml), TPOAb 30.6 IU/mL (0 IU/ml–60.0 IU/mL), IgG4 0.27 g/L (0.03 g/L–2.01 g/L), and serum amylase levels 63 U/L (35 U/L–135 U/L).

Based on the documented episodes of hypoglycemia, the inappropriately suppressed insulin and C-peptide levels, the pancreatic lesion on contrast-enhanced CT, the positive SACST, and exclusion of other differential diagnoses, a preliminary diagnosis of insulinoma was made. Since no obvious hyperparathyroidism or pituitary tumor was found, the patient’s solitary insulinoma was considered not to be associated with MEN-1.

## Surgical treatment and outcome

The patient underwent successful laparoscopic pancreatic tumor excision. Intraoperative adhesions were found in the abdominal cavity. A mass was identified in the body and tail of the pancreas, measuring 1.5 cm in diameter having a firm texture (**Figure 3**). The SACST helped determine the exact location of the functional lesion. This helped reduce surgical time and narrow the excision volume, thereby minimize excessive loss of normal pancreatic tissue, reducing the risk of postsurgical endocrine pancreatic insufficiency, and minimizing surgical trauma to the patient.

**Figure 3:**
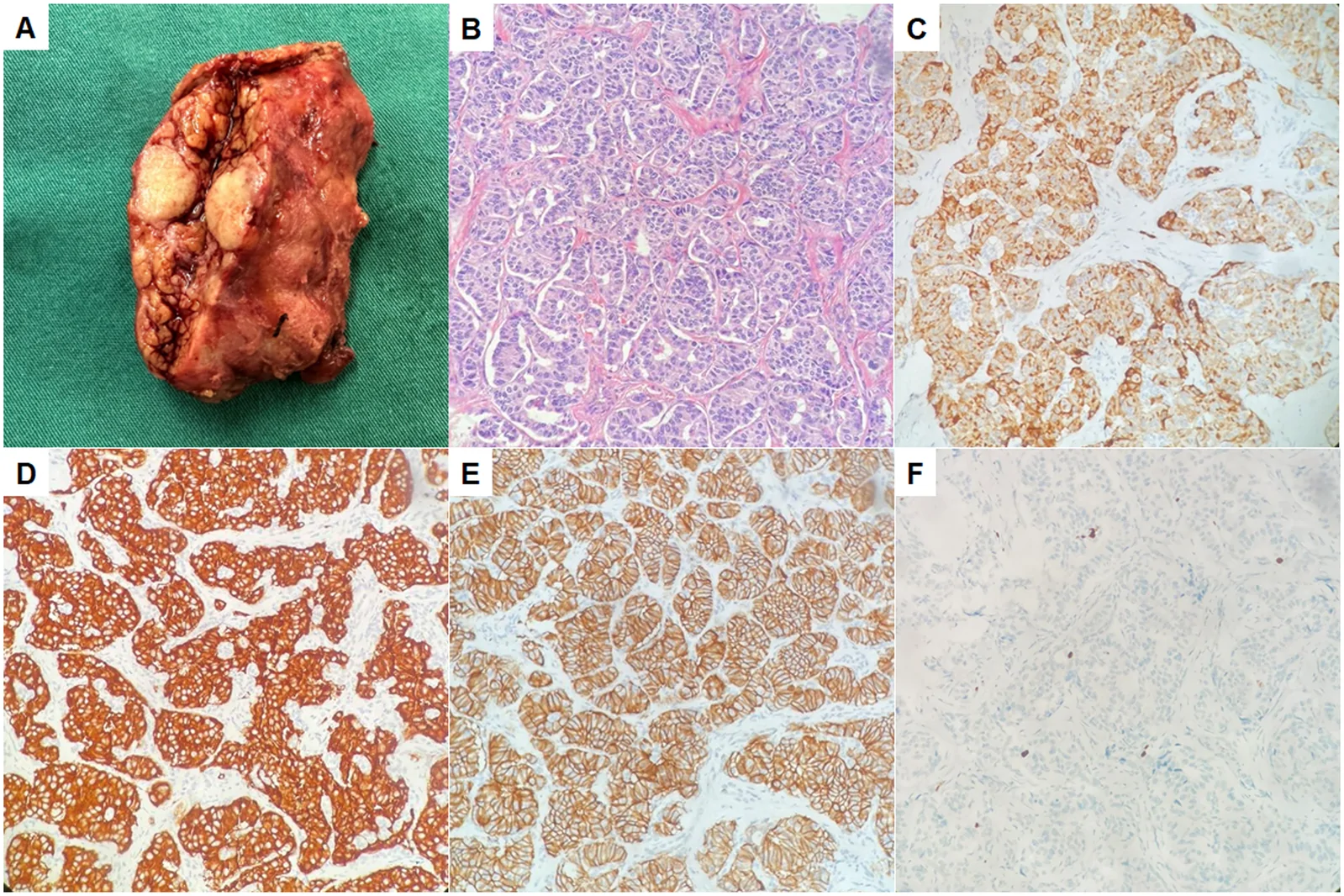
Surgical mass and histopathological staining. **(A)** A solitary 1.5 cm tumor with clean margins was resected. **(B)** H&E staining showed oval cells arranged in nested or trabecular patterns, with positive **(C)** chromogranin A, **(D)** synaptophysin, and **(E)** CD56, and 1% Ki-67 staining **(F)**.

Intraoperative frozen section indicated a neuroendocrine tumor with an intact capsule, and the surgical margin was negative.

Postoperative pathology was consistent with a neuroendocrine tumor (tumor grade G1), with a maximum tumor diameter of 1.2 cm, and the surgical margin was not involved. Immunohistochemical staining showed cytokeratin (CK) (partially weak +), chromogranin A (CgA) (+), synaptophysin (Syn) (+), CD56 (+), and Ki-67 (+) at 1% (**Figure 3**). A pathological diagnosis of insulinoma requires insulin staining, but insulin staining was not available for this case. Hence, the diagnosis of insulinoma was made based on the functional tests.

There were no hypoglycemic episodes postoperatively. On postoperative day 12, there was no fasting hypoglycemia (blood glucose level, 4.95 mmol/L) and a significant decrease in fasting blood insulin levels from 15.26 uIU/mL preoperatively to 2.54 uIU/mL postoperatively. Although endocrine pancreatic insufficiency is known to occur in less than 5% of patients who undergo central pancreatectomy (19), this is not suspected in this patient since the pancreatic insulin-secreting capacity seems to be preserved.

The patient received a final diagnosis of a well-differentiated, low-grade (G1) insulin-producing pancreatic neuroendocrine tumor, stage pT1.

## Discussion and literature review

Here we present a case of recurrent asymptomatic hypoglycemia with unremarkable contrast-enhanced CT and MRI imaging that led to delayed identification of insulinoma. The tumor was localized with the invasive selective arterial calcium stimulation test (SACST). There are several takeaways from this case.

First, when hypoglycemia occurs, a systematic, standardized protocol is needed to determine its cause. The 72-hour fasting test is considered the gold standard for evaluating hypoglycemia, with a sensitivity of 88.9% and a specificity of 100% for the diagnosis of insulinoma (20). However, in this case, the patient did not need to complete the full 72-h fasting test, as an episode of hypoglycemia due to hyperinsulinemia with elevated C-peptide levels was captured prior to the start of the test. It is important to measure blood glucose, insulin, and C-peptide levels concurrently during a hypoglycemia episode. Medical practitioners have proposed a shorter duration of the supervised fasting test or an overnight fast that has demonstrated high sensitivity within 24 h (**Table 3**). In some cases, the 72-hour test is recommended to reduce the risk of false-negatives results (21).

**Table 3:** Sensitivity of fasting test for endogenous hyperinsulinemia.

Author, Year	No. of patients	No. of patient with hypoglycemic event within fasting duration			
		Within <24 h	Within 24 h	Within 48 h	Within 72 h
Laroche, 1968 (22)	108	32 (30%) within 12 h	71 (66%)	102 (94%)	106 (98%)
Service, 1976 (23)	51	NA	NA	47 (92%)	NA
Service, 2000 (21)	170	33% within 12 h	65%	36 h in 84% within 36 h; 93% within 48 h	99%
Hirshberg, 2000 (24)	127	44 (42.5%) within 12 h	85 (66.9%)	120 (94.5%)	127 (100%)
Frajans, 1989 (25)	82	46 (56%) within 14 h; 64 (78%) within 18 h	71 (87%)	79 (96%)	82 (100%)
Vezzosi, 2007 (26)	33	27 (81.8%) within 12 h	31 (93.9%)	33 (100.0%)	NA
Mikovic, 2024 (27)	12	10 (83.3%) within 14 h	11 (91.7%)	12 (100%)	NA
Van Bon, 2009 (28)	9	1 (11.1%) within 12 h	4 (44.4%)	7 (77.8%)	9 (100%)
González-Vidal, 2025 (29)	10	3 (30%) within 12 h	8 (80%)	10 (100%) within 36 h	NA

Second, when the patient is diagnosed with hyperinsulinemic hypoglycemia, negative findings in initial anatomical lesion detection on CT and MRI do not exclude the possibility of intrapancreatic insulinoma. Most insulinomas are less than 2 cm (4, 10, 11). Manta et al. demonstrated that CT failed to detect 68.4% of lesions smaller than 1 cm and failed to detect more than 83% of lesions smaller than 2 cm (30). A number of studies carried out on the diagnostic accuracy of CT showed lesion detection rates ranging between 69% and 94% depending on tumor size and vascularization (31). Due to their hypervascularization, the arterial phase, with a sensitivity of 83%–88%, shows a clear advantage over the venous phase (sensitivity 11%–76%), especially in small tumors, such as insulinomas (31, 32). Another high-quality imaging modality essential for insulinoma localization is contrast-enhanced MRI, including diffusion-weighted imaging (DWI) with a sensitivity of 79% for tumors greater than 2.5 cm (19, 33). In our patient’s case, we repeatedly communicated with the nuclear medicine department and radiology department after negative findings on both CT and MRI. Although, a suspicious subcentimeter lesion was retrospectively seen on the arterial phase and DWI scans, it was not a definitive finding. In accordance with the current guidelines (4), if the initial CT is negative, the subsequent localization modality is endoscopic ultrasound (EUS) or MRI. More advanced imaging techniques, radiolabeled somatostatin receptor 2 (SSTR2) or glucagon-like peptide 1 receptor (GLP-1R) PET/CT or PET/MRI, especially ^68^Ga-DOTA-exendin-4 PET/CT, are also highly sensitive (4, 34, 35), but have limited availability or high cost. Unfortunately, radiolabeled SSTR2 and GLP-1R imaging are not available at our hospital. While both EUS with biopsy and SACST are invasive procedures, SACST exhibits higher sensitivity compared to EUS (sensitivity 93% versus 81%) (4, 34, 36). The sensitivity of EUS for detecting lesions in the pancreatic tail is significantly lower than that for lesions in the pancreatic head (37). Additionally, factors such as a low body mass index, female gender, young age, and the operator’s experience have a considerable influence on EUS outcomes (38). Consequently, for pancreatic tumors that remain undetected and whose functionality is uncertain, the SACT examination may offer greater advantages. In our case, the patient was female, and neither CT nor MRI provided a definitive localization; thus, we opted for a more cost-effective and functional test that can directly guide the next step in surgical treatment. Ultimately, it was confirmed that the tumor is situated in the tail of the pancreas, a region likely to evade EUS detection.

Third, in the case of negative imaging results in hyperinsulinemic hypoglycemia, the invasive selective arterial calcium stimulation test (SACST) can help determine whether insulinoma is intrapancreatic or extrapancreatic, and, if intrapancreatic, help guide surgical intervention for resection of the pancreatic region. The approach to solitary insulinomas differs from that of MEN1-associated insulinomas (4). In patients with confirmed endogenous hyperinsulinism, selective intra-arterial injection of calcium with sampling of hepatic vein insulin can regionalize the functional insulinoma and differentiate it from non-insulin-secreting pancreatic neuroendocrine tumors (4). Although this procedure is invasive, it has the potential to regionalize the insulinoma and aid in surgical exploration by limiting the extent of the resection, limiting loss of normal pancreatic tissue, minimizing intraoperative bleeding, and limiting procedure time for surgical exploration. The rationale for the SACST is that injection of calcium gluconate into the gastroduodenal, splenic, and superior mesenteric arteries stimulates insulin release from hyperfunctioning beta cells (insulinoma or pancreatic islet hyperplasia) but not from normal beta cells. The elevation of insulin levels detected from hepatic venous sampling regionalizes the source of abnormal beta cells (i.e., insulinoma) (39, 40). Multiple studies show a sensitivity greater than 90% for insulinoma (41–43) and can also distinguish it from non-insulinoma pancreatogenous hypoglycemia syndrome (NIPHS) by the latter’s moderate and diffuse response (44, 45). Using maximum increase in hepatic venous insulin concentration over baseline after calcium injection, cutpoints of >91.5 mIU/L and >263.5 mIU/L were 95% and 100% specific for insulinoma, respectively. In addition, a 19-fold increase in hepatic venous insulin over baseline was 99% specific for insulinoma. From the patient’s perspective, while SACST can accurately locate tumors and minimize surgical trauma, patients may experience heightened fear when confronted with invasive examinations compared to non-invasive options. This situation may necessitate thorough explanations from the physician or the consideration of alternative examination plans.

## Conclusion

For patients with a lesion diameter of less than 2 cm, like the patient in this case, symptoms are not typical, and active treatment is given after a clear diagnosis, and the postoperative recovery is good and the long-term benefit is great. This successful case also encourages us to be more confident in actively diagnosing functional cases with small lesions, especially cases where the initial imaging diagnosis is not clear.
